# News Items

**Published:** 1885-11

**Authors:** 


### NEWS ITEMS.

RUSH MEDICAL COLLEGE.—The winter session of the RUSH MEDICAL COLLEGE commenced on Tuesday evening, 22nd September, with an introductory address upon the subject of "Personal Identity," by PROFESSOR DE LASKIE MILLER. The term opened with 354 matriculates. The total number of students, registered at the present time, is 406.

CHICAGO MEDICAL COLLEGE.—PROFESSOR JOHN H. HOLLISTER delivered the introductory address at the opening of the winter term of the CHICAGO MEDICAL COLLEGE, Tuesday evening, 22nd September. PROFESSOR HOLLISTER sketched the life and labors of Louis Pasteur in terse, epigrammatic sentences.

During the absence of Dr. Frank Billings in Europe, Dr. Elbert Wing will deliver the course of lectures on physical diagnosis. Dr. Wing has recently returned from Vienna.

The total number of students, registered up to the present time, is 120.

College of Physicians and Surgeons.—The session of the College of Physicians and Surgeons of Chicago, for 1885–6, was opened on Friday evening, 25th September, with an introductory address on the subject of medical ethics and the code question, by Professor Oscar A. King.

Dr. Christian Fenger has been elected Professor of Clinical Surgery. Dr. N. Senn occupies the chair of Principles and Practice of Surgery and Clinical Surgery. Mr. C. B. Gibson has been chosen Professor of Inorganic Chemistry. Dr. C. A. Kelsey has received the chair of General Pathology and Pathological Anatomy. Dr. F. B. Earle has been appointed Curator of the Museum, and Dr. B. W. Rogers Assistant Demonstrator of Anatomy.

The following resignations from the faculty have been received and accepted: Dr. F. B. E. Bockius, chair of Medical Jurisprudence; Dr. W. A. Tohn, chair of Inorganic Chemistry; Dr. J. J. M. Angear, chair of the Principles of Medicine.

A College Hospital has been opened in the College building, and has proved a great success. Twenty beds are now occupied, and the Hospital Committee is about to add additional wards. Fifty beds will be the maximum capacity of the Hospital.

The total number of students in attendance at the present time is 160. The senior class has 65 members.

DR. CHARLES GILMAN SMITH has been elected Orator for 1886 by the ALUMNI ASSOCIATION of the MEDICAL DEPARTMENT of the UNIVERSITY of PENNSYLVANIA. DR. SMITH is a member of the Class of 1851.

COOK COUNTY HOSPITAL.—The investigation by the Hospital Committee of the County Board into the recently preferred charges of neglect of patients by the resident and visiting physicians at the COOK COUNTY HOSPITAL commenced Wednesday afternoon, 28th October. Doctors Adams, Hutchins, Robinson, Mitchell, Streeter, Tooker, Sherry, Burnside, McWilliams, Bridge, Caldwell, Rowan, St. John, Jacobson, Pratt, Holmes, and Dr. Andrews of Mercy Hospital were present. Commissioners Leyden, Hannigan, Lynn, Klehm, McCarthy, Senne, Nieson, McClaughrey, Ochs, and Van Pelt represented the Hospital Committee.

The investigation was instigated by charges of neglect, preferred by a female patient, suffering from necrosis of certain of the cranial bones, which was complicated by an abscess under the scalp, from which there was a continuous discharge of pus.

After the history of the patient's condition, prior to admission into the County Hospital, had been clearly narrated by Dr. Andrews, of Mercy Hospital, certain members of the Hospital Committee worked themselves up into a state of righteous fury without inquiring into the merits of the case. The lie direct was given and returned with the utmost prodigality in the matter of expletives, and the Committee adjourned in confusion.

During the meeting, Warden McGarigle made an interesting statement with reference to the obstetrical department, which, we are reliably informed, is true. During the past year three hundred and twenty-nine pregnant women have been received. Two hundred and fifty-eight births have occurred with only one maternal death.

The cost of supporting patients at the Cook County Hospital is seventy-six cents *per diem*, *per caput*,—less than in any other similar institution in the country.

The buildings, belonging to the County Hospital, are new, commodious, and supposed to be constructed in accordance with modern principle of hospital architecture.

The Resident Staff is annually selected with great care by competitive examination. The Visiting Staff includes certain physicians and surgeons, whose reputation is national. The nurses are supplied by the Cook County Training School. It is not necessary to speak of the merits of this institution to medical practitioners. Every one concedes that it is one of the most efficient organization of its kind in the United States.

Notwithstanding these facts, the Cook County Hospital is a political machine. Abuses in the management have been frequently detected.

It would not be a difficult matter to eliminate the factor, which has up to the present time handicapped the strenuous efforts of the medical attendants. A similar state of affairs recently existed in Blockley Hospital, attached to the Philadelphia almshouse.

The Committee of One-hundred effected a radical change by the ejection of the Board of Managers. The duty of the Citizen's Association of Chicago is apparent.

